# Breast cancer risk for women with diabetes and the impact of metformin: A meta‐analysis

**DOI:** 10.1002/cam4.5545

**Published:** 2022-12-19

**Authors:** Yifan Lu, Ali Hajjar, Vincent L. Cryns, Amy Trentham‐Dietz, Ronald E. Gangnon, Brandy M. Heckman‐Stoddard, Oguzhan Alagoz

**Affiliations:** ^1^ Department of Industrial and Systems Engineering University of Wisconsin‐Madison Madison Wisconsin USA; ^2^ Massachusetts General Hospital Institute for Technology Assessment, Harvard Medical School Boston Massachusetts USA; ^3^ Department of Medicine University of Wisconsin Carbone Cancer Center, University of Wisconsin‐Madison Madison Wisconsin USA; ^4^ Department of Population Health Sciences and the Carbone Cancer Center School of Medicine and Public Health, University of Wisconsin‐Madison Madison Wisconsin USA; ^5^ Departments of Biostatistics & Medical Informatics and Population Health Sciences University of Wisconsin‐Madison Madison Wisconsin USA; ^6^ Division of Cancer Prevention of the National Cancer Institute Bethesda Maryland USA; ^7^ Department of Industrial and Systems Engineering and Department of Population Health Sciences University of Wisconsin‐Madison Madison Wisconsin USA

**Keywords:** breast cancer, diabetes, meta‐analysis, metformin, type 2 diabetes

## Abstract

**Background:**

Diabetes mellitus has been associated with increased breast cancer (BC) risk; however, the magnitude of this effect is uncertain. This study focused on BC risk for women with type 2 diabetes mellitus (T2DM).

**Methods:**

Two separate meta‐analyses were conducted (1) to estimate the relative risk (RR) of BC for women with T2DM and (2) to evaluate the risk of BC for women with T2DM associated with the use of metformin, a common diabetes treatment. In addition, subgroup analyses adjusting for obesity as measured by body mass index (BMI) and menopausal status were also performed. Studies were identified via PubMed/Scopus database and manual search through April 2021.

**Results:**

A total of 30 and 15 studies were included in the first and second meta‐analyses, respectively. The summary RR of BC for women with T2DM was 1.15 (95% confidence interval [CI], 1.09–1.21). The subgroup analyses adjusting BMI and adjusting BMI and menopause resulted in a summary RR of 1.22 (95% CI, 1.15–1.30) and 1.20 (95% CI, 1.05–1.36), respectively. For women with T2DM, the summary RR of BC was 0.82 (95% CI, 0.60–1.12) for metformin users compared with nonmetformin users.

**Conclusions:**

Women with T2DM were more likely to be diagnosed with BC and this association was strengthened by adjusting for BMI and menopausal status. No statistically significant reduction of BC risk was observed among metformin users.

**Impact:**

These two meta‐analyses can inform decision‐making for women with type 2 diabetes regarding their use of metformin and the use of screening mammography for early detection of breast cancer.

## Graphical abstract

Relative risk of breast cancer among women with type 2 diabetes (CI, confidence interval; IV, inverse variance; SE, standard error).

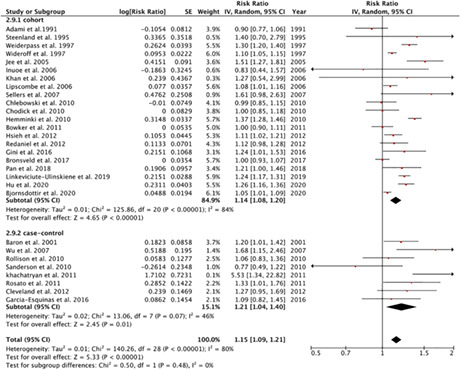

## 1 INTRODUCTION

Over 15 million American women have type 2 diabetes mellitus (hereafter referred to as type 2 diabetes) and this figure is projected to increase in the future.^1^ Type 2 diabetes increases the risk of other diseases including nephropathy, cardiovascular diseases, and retinopathy as well as cancer.^2^, ^3^, ^4^, ^5^ In particular, diabetes is recognized as an independent risk factor for breast cancer.^6^, ^7^, ^8^, ^9^

Many potential pathways have been suggested to explain the association between type 2 diabetes and breast cancer risk. For instance, type 2 diabetes is a chronic inflammatory disorder and is associated with inflammatory cell infiltrations, commonly seen in adipose tissue, which might lead to an increase in aromatase expression and increased local estrogen production. Increased estrogen production may drive the growth of estrogen receptor‐positive (ER+) breast cancer.^10^ Another biological link is hyperinsulinemia, a condition that occurs due to the body's resistance to the effects of insulin in the blood and the pancreas attempts to compensate for the lack of insulin by producing increasingly more insulin. Hyperinsulinemia is a risk factor for type 2 diabetes.^11^ Independent of obesity, alcohol consumption, physical inactivity, family history of breast cancer, history of benign breast disease, reproductive factors, and age, insulin resistance and hyperinsulinemia which are highly associated with diabetes have been identified as risk factors for breast cancer.^6^, ^7^, ^8^, ^9^

Three peer‐reviewed meta‐analysis studies, all published prior to 2012, examined the relationship between breast cancer risk and diabetes (type 1, type 2, or gestational diabetes). All three studies found that women with diabetes had an increased risk of breast cancer (reported relative risk [RR]: 1.20, 1.23, and 1.27).^12^, ^13^, ^14^

The objective of the first part of the present study is to estimate the risk of breast cancer only for women with type 2 diabetes. The objective of the second meta‐analysis in the present study is to estimate the breast cancer risk for women with type 2 diabetes associated with metformin use. Subgroup analyses with a focus on menopausal status and obesity had been planned depending on the availability of data.^15^, ^16^ A total of eight new studies published since prior meta‐analyses were included in our analysis. Moreover, while previous meta‐analyses included all types of diabetes including type 1 and type 2 diabetes, we focused solely on type 2 diabetes to ensure that our results are not confounded. This is crucial because type 1 diabetes is caused by autoimmune destruction of the islets and resulting insulin deficiency,^17^ whereas type 2 diabetes is linked to insulin resistance, inflammation, and high insulin levels,^18^ which drive the initiation and progression of cancers.^6^, ^8^, ^9^

The objective of the second meta‐analysis in the present study is to estimate the breast cancer risk for women with type 2 diabetes associated with metformin use. Subgroup analyses with a focus on menopausal status and obesity had been planned depending on the availability of data. Metformin is commonly prescribed to adults with type 2 diabetes, and around 40% of adults with type 2 diabetes in the USA take metformin as a treatment.^19^ Metformin has been suggested to inhibit cellular proliferation and tumor growth.^20^, ^21^, ^22^ Combined, these two meta‐analyses can inform decision‐making for women with type 2 diabetes regarding their use of metformin and the use of screening mammography for early detection of breast cancer.

## 2 MATERIALS AND METHODS

Following the guidance in the Cochrane Handbook,^23^ we estimated the RR for breast cancer for women with type 2 diabetes compared with those without diabetes and the RR for breast cancer for women with type 2 diabetes associated with metformin use. Subgroup analyses with a focus on menopausal status and obesity were planned depending on the availability of the data.

### 2.1 Search strategy

We searched the PubMed and Scopus databases through April 2021 for studies published in English using the search terms summarized in Table S1. Additional studies were identified through a manual search of references of review papers^12^, ^13^, ^14^, ^24^, ^25^ published after 2010.

### 2.2 Study selection

The meta‐analysis for the RR for breast cancer for women with type 2 diabetes compared with women without diabetes included (1) any observational study reporting an odds ratio (OR), RR, or hazard ratio (HR) estimate with a 95% confidence interval (CI), and (2) for females diagnosed with type 2 diabetes (or diagnosed at age ≥30 if the type of diabetes was not specified). We used 30 as an age cutoff which is a common age cutoff to distinguish type 2 from type 1 diabetes in previous studies.^26^, ^27^, ^28^

The meta‐analysis for the RR of breast cancer for women with type 2 diabetes associated with metformin use included (1) any observational study reporting an OR, RR, or HR estimate with 95% CI, (2) comparing metformin users to average nonmetformin users, and (3) included studies of females diagnosed with type 2 diabetes (or diagnosed at age ≥30 if the type of diabetes was not specified).

Two independent investigators (Y.L. and A.H.) identified eligible studies by screening titles and abstracts. Disagreements related to the selection of the studies were resolved by a consensus decision after discussion.

### 2.3 Data extraction and quality assessment

Two investigators (Y.L. and A.H.) independently reviewed the selected studies and extracted the relevant information including full citation (authors, year of publication), geographic location of participant recruitment, age of the study population, adjustment of covariates, risk estimate, and the corresponding 95% CI. When one study reported multiple HRs, RRs, or ORs, the estimate adjusted for the highest number of covariates (first priority) for the longest duration of follow‐up was used (second priority). When multiple publications used the same cohort, only the result from the most recent publication was included, and if the previous publications reported the results by adjusting for different sets of covariates, they were used in the subgroup analysis.

Two investigators (Y.L. and A.H.) independently evaluated the study quality using the Newcastle‐Ottawa Assessment scale, an assessment for observational studies, in terms of selection, comparability, and exposure for cohort studies and selection, comparability, and outcomes for case–control studies.^29^ Each study was assigned a score ranging from 0 to 9 with higher scores indicating higher quality. Each cohort study was judged on four items related to selection (representativeness of exposed cohort, selection of nonexposed cohort, ascertainment of exposure, and demonstration of outcome not presented at start), one item related to comparability (control for other confounding factors), and three items related to exposure (assessment of outcome, enough length for follow‐up and adequacy of follow‐up cohorts). A cohort study could be assigned one point for each item in selection and exposure categories and two points in comparability. Each case–control study was judged on four items related to selection (definition of cases, definition of controls representativeness of the cases, and selection of controls), one item related to comparability (control for other confounding factors), and three items related to outcome (assessment of outcome, same analysis method for cases and controls, and nonresponse rates). A case–control study could be assigned one point for each item in the selection and outcome categories and two points in comparability.^29^

### 2.4 Statistical analysis

All extracted data were analyzed by Revman5 and MedCalc.^30^, ^31^ Summary risk estimates were obtained using a random effects model with the inverse variance method. The $I^{2}$ test was used to evaluate heterogeneity across studies, where the recommended categories are set as possibly not important for $I^{2}$ = 0%–40%; moderate for $I^{2}$ = 30%–60%; substantial for $I^{2}$ = 50%–90%; and considerable for $I^{2}$ = 70%–100%.^23^ Subgroup analyses were performed for further investigation of the RR for women with type 2 diabetes. Two subgroups were created to investigate the factors of obesity and menopausal status. Furthermore, we summarized the estimates for pre‐ and postmenopausal women separately. When the studies included estimates stratified by menopausal status, we used those estimates for subgroup analysis instead of the overall estimates. Funnel plots along with Egger's test were used to assess potential publication bias. An asymmetric funnel plot will indicate a risk of publication bias, and this is further verified by Egger's test with a *p* value of <0.05.^23^, ^26^, ^32^

## 3 RESULTS

### 3.1 The relative risk of breast cancer according to type 2 diabetes

**FIGURE 1:**
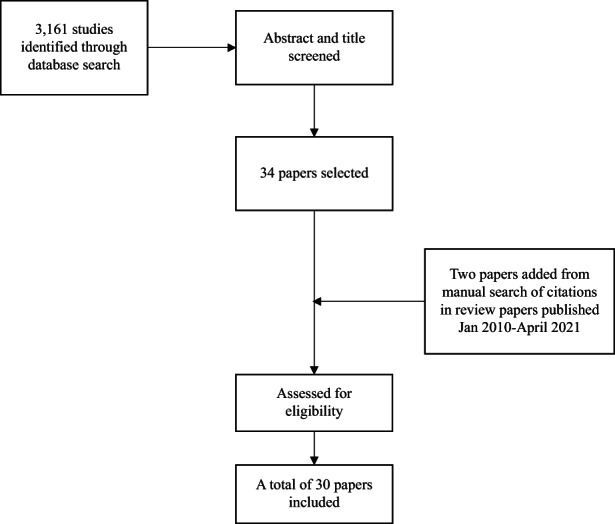
Flow chart of the literature search process for the meta‐analysis estimating the relative risk of breast cancer for women with type 2 diabetes

Of 3,161 possible studies identified through database search, 34 relevant studies of the effect of type 2 diabetes on breast cancer were found after screening abstracts and titles, and any nonobservational study was excluded during this process. Two additional studies^33^, ^34^ were found through a manual search of references in other review papers.^12^, ^13^, ^14^ After assessing the eligibility of each study according to our inclusion criteria, we included a total of 30 studies for analysis. Among the 30 studies, two studies^35^, ^36^ used the same cohort. Since one of them adjusted for menopausal status and BMI,^36^ we used one of them in estimating the overall effect, and the other one in the subgroup analysis. Figure 1 describes the screening and selection process. The characteristics of each study were summarized in Table 1.

**TABLE 1:** Characteristics of the studies included in the meta‐analysis estimating the relative risk of breast cancer for women with type 2 diabetes

Study	Geographic location	Study design	No. of cases	No. of controls	Age	RR/OR/HR	95% CI	Adjustment	Quality score
Adami et al.^34^	Sweden	Cohort	27,862	N/A	≥20	0.9	0.8–1.1	Age and sex	6
Steenland et al.^37^	USA	Cohort	1,250	11,804	Avg = 60	1.4	0.7–2.78	Age, BMI, smoking, alcohol, income, physical activity, and menopausal status	8
Weiderpass et al.^38^	Sweden	Cohort	70,110	N/A	≥40	1.3	1.2–1.4	Age and sex	6
Wideroff et al.^39^	Denmark	Cohort	55,010	N/A	≥50	1.1	1.1–1.2	Age and sex	7
Baron et al.^40^	USA	Case–control	5,659	5,928	50–75	1.2	1–1.4	Age, state, age at first birth, BMI, family history of breast cancer, HRT use, menopausal status, age at menopause, OC use, parity, alcohol use	6
Jee et al.^41^	Korea	Cohort	21,056	270,157	30–95	1.51	1.26–1.8	Age, age squared, amount of smoking, and alcohol use	6
Inoue et al.^42^	Japan	Cohort	1,571	49,652	40–69	0.83	0.44–1.57	Age at baseline, study area, history of cerebrovascular disease, history of ischemic heart disease, smoking, ethanol intake, body mass index, leisure‐time physical activity, green vegetable intake, and coffee intake	6
Khan et al.^43^	Japan	Cohort	1,544	31,949	40–79	1.27	0.51–3.14	Age, BMI, smoking, and drinking	4
Lipscombe et al.^44^	Canada	Cohort	73,796	391,714	55–59	1.08	1.00–1.15	Age	7
Sellers et al.^45^	US	Cohort	403	5,725	≥18, Avg = 47	1.61	0.98–2.62	Age, BMI, family history of breast cancer, parity education, alcohol use, physical activity, oral contraceptive use, age at menarche, hormone replacement therapy	4
Wu et al.^46^	USA	Case–control	1,248	1,148	25–74	1.68	1.15–2.47	Age, BMI, menopausal status, race, and education	6
Rollison et al.^47^	USA	Case–control	330	2,193	≥15	1.06	0.8–1.32	Age at menopause, use of postmenopausal hormones, physical activity, and alcohol consumption	7
Hemminki et al.^48^	Sweden	Cohort	125,126	922,796	≥39	1.37	1.28–1.46	Age and sex	5
Chodick et al.^49^	Israel	Cohort	16,721	83,874	≥21, avg = 61	1	0.86–1.19	Age, region, SES level, use of healthcare services a year prior or index date, BMI, and history of cardiovascular diseases	6
Sanderson et al.^50^	USA	Case–control	190	468	30–79	0.77	0.49–1.23	Menopausal status and mammography screening	5
Bowker et al.^51^	Canada	Cohort	84,506	84,506	Avg = 61.8	1	0.9–1.11	Age, social economic status, number of physician visits, and year of diagnosis	6
Khachatryan et al.^33^	Armenia	Case–control	184	184	35–70	5.53	1.34–22.81	Age, BMI, Age at menarche, Age at 1st pregnancy, live birth, abortion, breastfeeding duration, age at menopause, hormone replacement therapy	3
Rosato et al.^52^	Italy and Swiss	Case–control	3,869	4,082	33–80	1.33	1.3–2.27	Age, study center study period, education, alcohol consumption, age at menarche, age at first birth, age at menopause, hormone replacement therapy use, and family history of Breast cancer	7
Redaniel et al.^53^	UK	Cohort	52,657	30,210	≥35	1.12	0.98–1.29	Age period, region, and BMI	8
Cleveland et al.^54^	USA	Case–control	1,495	1,453	≥30	1.27	0.95–1.69	Age, menopausal status, race, and BMI	6
Chlebowski et al.^55^	USA	Cohort	3,401	64,618	50–79	0.99	0.85–1.14	Age, first‐degree relative with breast cancer, benign breast disease, age at menarche, age at menopause, parity, age at first birth, education, number of months of breastfeeding, smoking, alcohol consumption, body mass index, physical activity, duration of use of estrogen alone, duration of use of estrogen plus progesterone, bilateral oophorectomy, and mammogram within 2 years of baseline; stratified according to age (10‐year groups), hormone therapy trial randomization, dietary trial randomization or overall survival enrollment, enrollment onto Women's Health Initiative extension, and race/ethnicity	6
Gini et al.^56^	Italy	Cohort	14,420	1,018,518	40–84	1.24	1.00–1.52	Age, sex, and year of cancer diagnosis	6
Garcia‐Esquinas et al.^57^	Spain	Case–control	916	1,094	20–85	1.09	0.82–1.45	Age, period, region, and BMI	8
Bronsveld et al.^58^	UK	Cohort	147,998	147,998	≥18	1	0.94–1.08	Age	7
Pan et al.^27^	China	Cohort	18,305	281,705	30–79	1.21	1.01–1.47	Age, region, education, patient history of cancer, BMI, cigarette smoking, alcohol drinking, and physical activity	7
Linkeviciute‐Ulinskiene et al.^59^	USA	Cohort	78,823	N/A	≥40	1.24	1.17–1.31	Age and sex	5
Hu et al.^35^	USA	Cohort	13,077	3,355,787	30–75	1.26	1.17–1.37	Age, ethnicity, smoking status, alcohol intake, multivitamin use, physical activity, total energy, alternative healthy eating index, family history of diabetes, family history of cancer, endoscopy screening, fasting glucose screening, insulin use, oral hypoglycemic drug use, mammography screening, postmenopausal hormone use, and oral contraceptive use, BMI	7

*Note*: Quality score based on Newcastle‐Ottawa Assessment scale.

Abbreviations: Avg, average; CI, confidence interval; HR, hazard ratio; OR, odds ratio.

The quality of the included studies was moderate with an average score of 6 using the Newcastle‐Ottawa Assessment scale. Of the 30 studies, 11 studies had high quality (scoring between 7 and 9); 16 studies had moderate quality (scoring between 5 and 6); and three studies had low quality (scoring below 4) (Table 1).

Considerable heterogeneity was observed among the included studies as the $I^{2}$ was 81%; and thus, the random effects model was used. A funnel plot was produced, and no obvious evidence of publication bias was observed as the *p* value was 0.34 from Egger's test (Figure S1).

**FIGURE 2:**
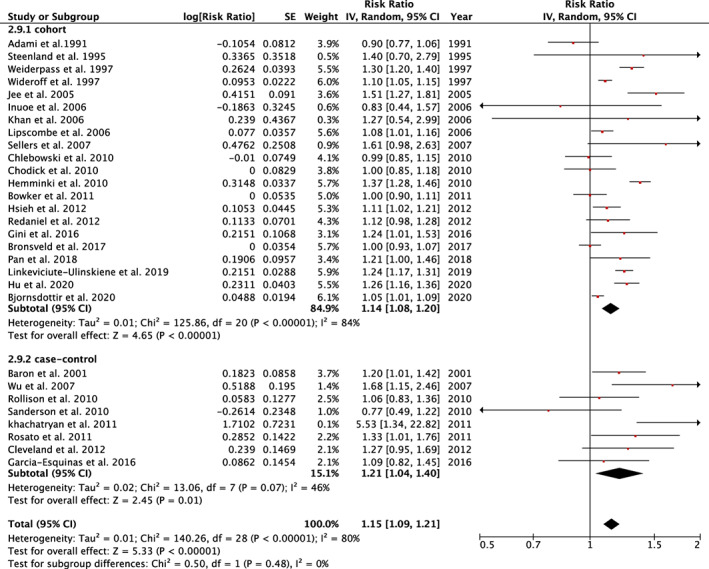
Summary of relative risk of breast cancer among women with type 2 diabetes according to study design (CI, confidence interval; IV, inverse variance; SE, standard error)

The overall effect indicated that women with type 2 diabetes were more likely to have breast cancer (RR = 1.15; 95% CI, 1.09–1.21). The summary risk estimate using only the cohort studies (RR = 1.14; 95% CI, 1.08–1.20) was consistent with that using only the case–control studies (RR = 1.21; 95% CI, 1.04–1.40) (Figure 2). Of the 29 studies included in this meta‐analysis, six studies were conducted in Asia^27^, ^33^, ^41^, ^42^, ^43^, ^60^ with a summary RR equal to 1.25 (95% CI, 1.03–1.52); two studies focused on Hispanic women^47^, ^50^; and one study focused on Asian‐American women.^46^ The summary RR was 1.13 (95% CI,1.07–1.20) for the rest of the studies conducted in Europe or North America (Figure S2).

A limited number of studies presented results according to race or ethnicity, and subgroup analyses were conducted using the 20 studies in which women were randomly sampled in European and North American countries. A slightly greater RR was observed among the seven studies that provided estimates adjusted for BMI (RR = 1.22; 95% CI, 1.15–1.30). For the three studies adjusting for BMI and menopausal status, the summary RR was 1.20 (95% CI, 1.05–1.36). Furthermore, some studies reported their estimates stratified by menopausal status. The summary RR was 1.07 (95% CI, 1.03–1.11) and 0.97 (95% CI, 0.88–1.07) for the risk of breast cancer associated with type 2 diabetes for post‐ and premenopausal women with diabetes, respectively (Table S2 and Figure S3).

### 3.2 The relative risk of breast cancer according to the use of metformin

**FIGURE 3:**
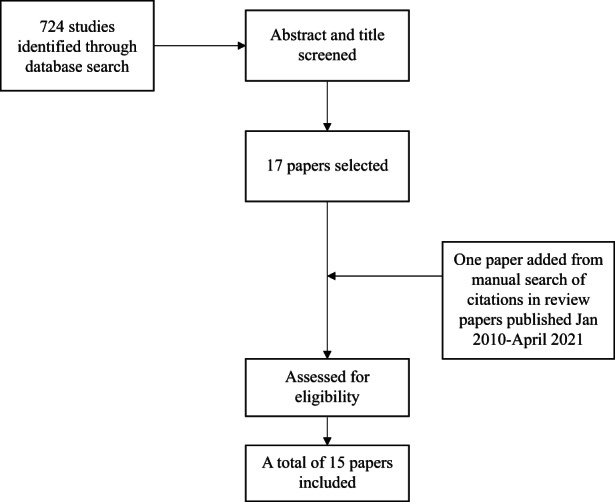
Flow chart of the literature search process for the meta‐analysis estimating the relative risk of breast cancer for women using metformin

For the second meta‐analysis, we identified 724 studies to estimate the risk of breast cancer according to metformin use for women with type 2 diabetes through a database search, and 17 studies were selected after screening abstracts and titles. One study^28^ was added through a manual search of the bibliographies of recent reviews.^28^, ^61^ After assessing the eligibility of each study, we included a total of 15 studies for the final analysis (Figure 3). The characteristics of each study were summarized in Table 2.

**TABLE 2:** Characteristics of the studies included in the meta‐analysis estimating the relative risk of breast cancer for women with type 2 diabetes who use metformin

Study	Geographic location	Study design	No. of cases	No. of controls	Age	RR/OR/HR	95% CI	Adjustment	Quality score
Libby et al.^28^	UK	Cohort	4,085	4,085	35–100	0.60	0.32–1.1	Age, smoking, deprivation, BMI, A1C, insulin use, and sulfonylurea use	6
Bodmer et al.^62^	UK	Case–control	305	1,153	30–79	0.42	0.21–0.77	Age, sex, general practice, and calendar time by matching and further adjusted for each other plus the use of prandial glucose regulators, acarbose, estrogens, smoking, BMI, diabetes duration, and A1C	8
Morden et al.^63^	USA	Cohort	15,286	66,395	≥68	1.28	1.05–1.57	N/A	6
Bosco et al.^64^	Denmark	Case–control	1,250	3,073	≥50	0.83	0.56–1.22	Confounding and selection bias were introduced by matching on the county, and complications due to diabetes, clinical obesity, age at index date, and postmenopausal hormone use	8
Chlebowski et al.^55^	USA	Cohort	3,401	64,618	50–79	0.75	0.57–0.99	Age, first‐degree relative with breast cancer, benign breast disease, age at menarche, age at menopause, parity, age at first birth, education, number of months of breastfeeding, smoking, alcohol consumption, body mass index, physical activity, duration of use of estrogen alone, duration of use of estrogen plus progesterone, bilateral oophorectomy, and mammogram within 2 years of baseline; stratified according to age (10‐year groups), hormone therapy trial randomization, dietary trial randomization or overall survival enrollment, enrollment onto Women's Health Initiative extension, and race/ethnicity	6
Redaniel et al.^53^	UK	Cohort	52,657	30,210	≥35	1.02	0.79–1.3	Age, region and BMI	8
Tseng et al.^65^	Taiwan	Cohort	191,195	285,087	Avg = 57	0.28	0.25–0.31	Age and obesity	8
Chen et al.^66^	Taiwan	Cohort	2,223	5,102	≥30	0.8	0.3–2.13	Age, sex, Charlson comorbidity index, smoking‐related comorbidities, alcohol use disorders, morbid obesity, pancreatitis, hypertension, monthly income, and urbanization level	7
Soffer et al.^67^	US	Cohort	4,887	61,891	18–103	1.08	0.88–1.32	Age of index diabetes diagnosis, race/ethnicity, ERT status, statin use, Charlson comorbidity index, and outpatient utilization	7
Garcia‐Esquinas et al.^57^	Spain	Case–control	916	1,094	20–85	0.95	0.86–1.04	Age, period, region, and BMI	8
Calip et al.^68^	US	Cohort	10,050	9,749	≥40	1.14	0.68–1.91	Other diabetes medications, age at cohort entry, study entry year, smoking status, menopausal status, Charlson score, and statin	8
Vicentini et al.^69^	Italy	Cohort	7,460	9,566	20–84	0.69	0.32–1.48	N/A	7
Hosio et al.^70^	Finland	Cohort	376,233	266,557	≥40	0.94	0.86–1.04	N/A	6
Dankner et al.^71^	Isreal	Cohort	172,948	142,942	21–87	0.88	0.56–1.39	Age, sex, socioeconomic status, ethnic origin, smoking and parity.	8
Sung et al.^72^	Hongkong	Cohort	11,365	22,730	≥18	1.32	0.88–1.97	Age, sex, and comorbidities	7

*Note*: Quality score based on Newcastle‐Ottawa Assessment scale.

Abbreviations: Avg, average; CI, confidence interval; HR, hazard ratio; OR, odds ratio.

Considerable heterogeneity was identified among studies as the $I^{2}$ was 97%. Therefore, the random effects model was used in the analysis. A funnel plot was produced, and no obvious evidence of publication bias was observed as the *p* value was 0.65 from Egger's test (Figure S4).

The quality of the included studies was high with an average score of 7 using the Newcastle‐Ottawa Assessment scale. Of the 15 studies, 11 studies had high quality, whereas four studies had moderate quality (Table 2).

**FIGURE 4:**
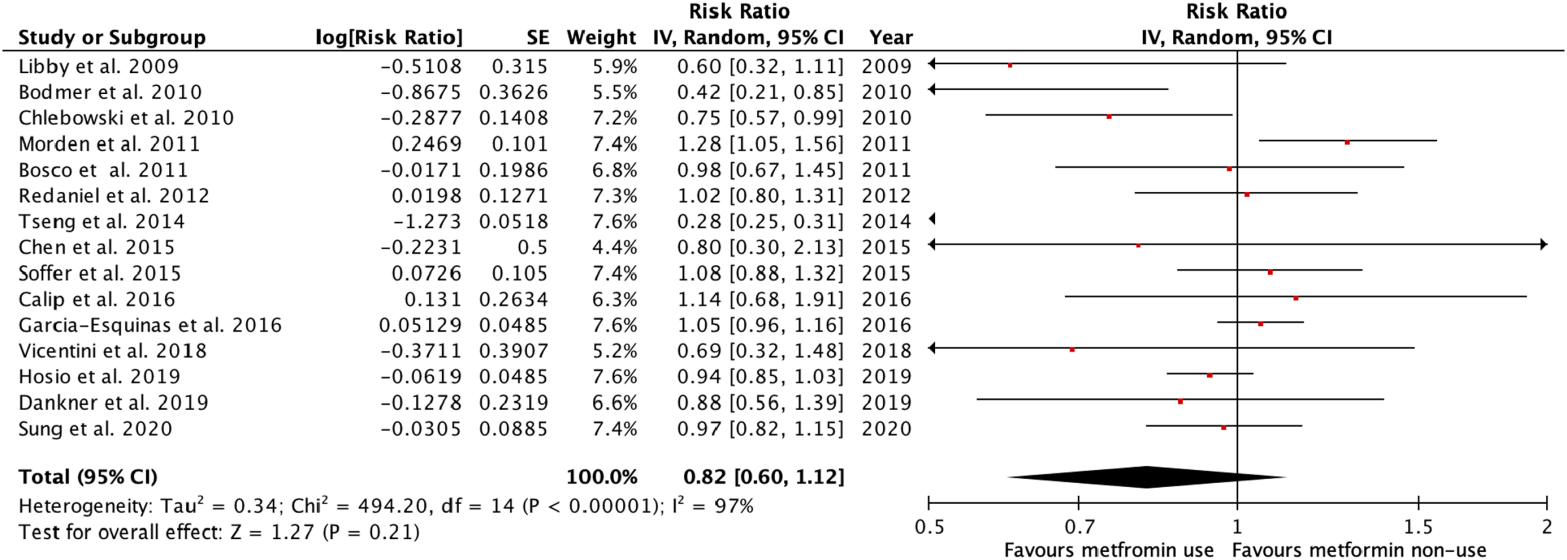
Summary of relative risk of breast cancer according to the use of metformin among women with type 2 diabetes (CI, confidence interval; IV, inverse variance; SE, standard error)

The overall risk estimate of breast cancer risk associated with the use of metformin was 0.82 with substantial variation around this estimate (95% CI, 0.60–1.12) (Figure 4). The limited number of studies prevented a subgroup analysis.

## 4 DISCUSSION

Our analysis found that women with type 2 diabetes were more likely to have breast cancer (RR = 1.15; 95% CI, 1.09–1.21). A stronger association was observed after the adjustment of BMI or BMI and menopausal status (RR = 1.22; 95% CI, 1.15–1.30 and 1.20; 95% CI, 1.05–1.35, respectively). For women with type 2 diabetes, our meta‐analysis suggested that metformin use was associated with a reduced risk of breast cancer although the variation around the risk estimate was large (RR = 0.82, 95% CI, 0.60–1.12).

We found three meta‐analysis studies on the relationship between breast cancer risk and diabetes published since 2010. Note that while none of the three previous reviews considered a quality assessment for the included studies, we assigned a score to each study using Newcastle‐Ottawa Assessment scale to represent its quality. This approach has led to a more reliable estimation of the RR. More specifically, three studies in our meta‐analysis were evaluated as low quality,^33^, ^43^, ^45^ and these three studies had the minor effect on the overall estimate. The summary RR of breast cancer for women with type 2 diabetes was 1.14 (95% CI, 1.09–1.20) when these three studies were excluded.^33^, ^43^, ^45^

The study published by Boyle et al.^12^ included 40 studies. They found women with diabetes had a higher risk of breast cancer (RR = 1.27; 95% CI, 1.16–1.39), and the study population included female patients having type 1 or type 2 diabetes. The meta‐analysis published by Hardefeldt et al.^14^ included 43 observational studies on female or male patients having type 1, type 2, or gestational diabetes. The summary RR was 1.20 (95% CI, 1.13–1.29), and evidence of publication bias was observed. These two studies included a larger number of studies compared to the present study since we focused on only type 2 diabetes, whereas they included studies focusing on all types of diabetes. The meta‐analysis published by Liao et al. in 2011 included a total of 12 observational studies, some of which did not specify the type of diabetes. The summary RR was 1.23 (95% CI, 1.18–1.27).^13^ In addition to these three peer‐reviewed publications, we found a conference presentation from Bota et al.,^73^ which reported a summary RR of 1.13 (95% CI, 1.04–1.24). No details on the selected studies were provided in this presentation.

The overall estimates on summary RR from the three meta‐analyses are higher than our summary RR estimate as we limited the study population to female patients with only type 2 diabetes. A subgroup analysis on women with only type 2 diabetes by Boyle et al.^12^ including 14 studies reported an RR of 1.16 (95% CI, 1.04–1.29), which is consistent with our estimate, and all of those 14 studies were included in our analysis. They also found that the association was stronger when only studies published after 1997 were included in the meta‐analysis.^12^ Another subgroup analysis on type 2 diabetes was conducted by Hardefeldt et al.,^13^ with 10 studies all published after 1997, reported an RR of 1.22 (95% CI, 1.07–1.40), and all these studies were included in our analysis. The trend was not obvious in our analysis as our analysis included only four studies published before 1997 (Figure S5). There are two possible reasons for this discrepancy. First, we included eight new studies^27^, ^35^, ^53^, ^56^, ^57^, ^59^, ^74^, ^75^ having smaller risk estimates with a summary RR of 1.14 (95% CI, 1.05–1.24) and we only included studies on women with type 2 diabetes. Second, the use of metformin may inhibit the growth of certain tumors.^22^ Metformin is the most widely used oral medication for diabetes since the 1950s, and the extended release of metformin was approved in the USA in 2000.^76^, ^77^, ^78^ In the US, around 40% of diabetes patients use metformin as a treatment.^19^ Thus, the wide use of metformin might have also compromised the effect of diabetes mellitus on breast cancer risk.

Boyle et al.^12^ and Liao et al.^13^ found postmenopausal women with diabetes had a 15% and 23% higher risk of breast cancer, respectively, while the association between diabetes and breast cancer risk was closer to the null for premenopausal women.^12^, ^13^ We observed the same trend in our analysis. Of the 20 studies, a total of 11 studies indicated the menopausal status of their study population, where five studies reported their estimates stratified by menopausal status and six studies were on postmenopausal women. The summary RR of breast cancer was 1.07 (95% CI, 1.03–1.11) and 0.94 (95% CI, 0.81–1.09) for post‐ and premenopausal women with diabetes, respectively (Table S2 and Figure S5). However, the determination for pre‐ and postmenopausal status varies among the studies we included. Some studies used simple age cutoff to separate pre‐ and postmenopausal groups, whereas others used menstrual data to define a woman as postmenopausal if she reported no cycles within 12 months.

Obesity is a confounding factor for the association between diabetes and the risk of breast cancer.^16^ In 2018, around 90% of patients with diabetes were overweight or obese in the USA.^1^ We conducted a subgroup analysis to account for the effect of obesity. Of the 20 studies in the meta‐analysis, seven studies adjusting breast cancer risk for BMI resulted in a summary RR of 1.22 (95% CI, 1.15–1.30). Boyle et al.^12^ and Hardefeldt et al.^14^ conducted a subgroup analysis with the studies that adjusted for BMI and found that the summary RR were 1.16 (95% CI, 1.08–1.24) and 1.12 (95% CI, 1.04–1.21), respectively. We observed a stronger association among the studies that adjusted for BMI. The association was also stronger than our overall estimate. In the present seven studies adjusting for BMI, six of them either included predominately postmenopausal women or had a study population with an average age over 50. Therefore, the study population in seven studies consisted of mostly older and postmenopausal women, leading to a higher RR in the subgroup analysis among the studies that included BMI. This is likely because studies of the risk of breast cancer and obesity have generally observed an increased risk of breast cancer for overweight and premenopausal women.^79^ Furthermore, as noted by the study by Boyle et al.,^12^ various methods of adjustment for BMI were used within the studies included in the meta‐analysis. Most of the studies that were included in our analysis did not state how they parameterized BMI for adjustment in their statistical model.

There are some limitations to our analysis. In addition to obesity, menopausal status is another factor that might affect the final estimate.^15^ In the subgroup analysis, although we suspect both BMI and menopausal status can impact the RR of breast cancer for women with type 2 diabetes, only three studies adjusted for BMI and menopausal status. Thus, the reliability of our estimate when both BMI and menopausal status were adjusted was compromised by the limited number of included studies. Considerable heterogeneity was observed among studies, and this might be largely caused by the differences in adjustment for confounding variables among studies. Furthermore, although no strong indication of publication bias was observed from the funnel plot and Egger's test, the actual RR might be higher than we observed as diabetes is still an under‐diagnosed disease.^1^

We also did not account for the differences in mammography screening utilization between women with and without diabetes. We do not expect the control on mammography screening utilization would affect our results. This is because although women with diabetes have higher healthcare utilization than women without diabetes, the complexity of diabetes care often decreases the rates of mammography screening for women with diabetes.^80^, ^81^, ^82^, ^83^, ^84^ For the 30 studies in our analysis, only three studies adjusted for the effect of mammography screening.^35^, ^50^, ^55^ Lack of control for mammography screening utilization may have led to the underestimation of the RR of cancer since women with diabetes who have existing breast cancer at the time of the study may not have been diagnosed due to lower rates of screening. This was further verified by the differences in the results from case–control studies and cohort studies. A stronger effect was observed among case–control studies, while cohort studies, especially those with long follow‐up, may be vulnerable to the screening bias and led to an overall lower estimate.

Two review studies examined the risk of breast cancer for women with type 2 diabetes associated with metformin use. The review by Tang et al.^24^included 25 estimates from 12 observational studies up to November 2016, which found no significant association between metformin exposure and risk of breast cancer (RR = 0.93; 95% CI, 0.85–1.03). Multiple estimates were included from some studies in this review as these studies included results for several different types of glucose‐lowering medicines. Of the total 12 studies, Currie et al.,^61^ Ruiter et al.,^85^ Hsieh et al.,^60^ Tsilidis et al.,^86^ and Kowall et al.^87^ were not included in the present study because their reference groups were sulfonylurea or insulin‐based treatment users, while we only considered nonmetformin users as comparators as we suspect other glucose‐lowering medicines may increase or decrease the risk of breast cancer.^88^ Tang et al.^24^ suggested the presence of publication bias based on their Egger's test.

Another study of the association between metformin use and breast cancer risk by Yang et al. included 11 estimates from nine studies, which reported a summary RR of 0.96 (95% CI, 0.76–1.22). Among the nine studies, Tsilidis et al.,^86^ Qiu et al.,^89^ and Hsieh et al.^60^ were not included because their reference groups were either sulfonylurea or insulin‐based treatment users. Three estimates from different durations of metformin use were included in the same study done by Bodmer et al. In this analysis, we only included the estimate for the longest duration of metformin use from Bodmer et al.^62^ Evidence of publication bias was also observed in this review article based on Egger's test.^25^

Our meta‐analysis included four additional studies published after 2016. However, similar to these studies, while we found a slightly lower summary RR (0.82; 95% CI, 0.60–1.12), the association was not statistically significant.

The summary estimate was highly driven by the estimate from Tseng et al.,^65^ which is a large‐scale study reporting the lowest HR with the smallest standard error and accounted for a weight of 7.6% in our analysis. After removing this study, the summary RR increased dramatically to 0.98 (95% CI, 0.89–1.07).

Unlike the previous reviews, no obvious evidence of publication bias among the studies was observed as the *p* value from Egger's test was 0.65. Although two prior reviews included estimates compared with other glucose‐lowering medicines (sulfonylurea or insulin‐based treatments), we only selected studies having nonmetformin users as the reference group. Furthermore, although Egger's test can be used to assess the asymmetry of funnel plots, it works better for continuous outcomes with intervention effects measured as mean differences.^23^ Therefore, other possible reasons might affect the reliability of our results. As discussed above, obesity and menopausal status could serve as two confounding factors that may have affected our results. Unfortunately, we could not conduct subgroup analyses with a focus on obesity and menopausal status due to the limited number of studies. We found the baseline characteristics of the included metformin users were different across the included studies. Differences existed among the inclusion criteria for the minimum dose and length of use, and a stronger association was found among the few studies with a longer duration of metformin use. Another limitation of our study is that we cannot make a definite conclusion on the required dose of metformin to decrease the risk of developing breast cancer. This is because the studies included in this meta‐analysis provide little information on the dose of metformin and therefore the minimum dose of metformin likely varies across the studies. Metformin, as a treatment for type 2 diabetes, lowers blood glucose levels, and research shows that the antitumor effect of metformin depends on both glucose availability and metformin concentration.^90^, ^91^ Among the studies we included, only two studies adjusted their results with hemoglobin A1C (a measure of glucose control) test results,^28^, ^62^ and the majority did not. The cohort study from Libby et al.^28^ included 4,085 cases and 4,085 controls, and the result did not show a statistically significant reduction of breast cancer risk associated with the use of metformin (RR = 0.60; 95% CI, 0.32–1.10). The case–control study from Bodmer et al.^62^ included 305 cases and 1,153 controls, and the result indicated a preventive effect of metformin on breast cancer (RR = 0.42; 95% CI, 0.21–0.77). The weights for these studies are relatively smaller compared with other studies in the calculation of the overall estimates because of their wide CIs. Therefore, we cannot make a definite conclusion based on these studies but suspect that blood glucose level could be another confounding factor affecting our estimates. In addition, patients who take metformin may also receive other glucose‐lowering medicines, and there is no information on the number of such patients in most of the studies included in our meta‐analysis, as other glucose‐lowering medicines can serve as a confounding factor affecting our estimate of the risk of breast cancer.^88^

Overall, obesity and menopausal status could serve as confounding factors that may have affected our results. However, a limited number of observational studies adjusted for these two factors, and thus, future work could examine the effects of these two factors. Also, based on the estimates from these two meta‐analyses, another future research direction could be to optimize the decision‐making for women with type 2 diabetes regarding their use of metformin and the use of screening mammography for early detection of breast cancer.

## 5 CONCLUSION

Overall, we found women with type 2 diabetes were about 15% more likely to be diagnosed with breast cancer than women without type 2 diabetes. A stronger association (RR = 1.22) was observed after the adjustment of BMI and menopausal status. Metformin use was not associated with a statistically significant reduction in breast cancer risk.

## AUTHOR CONTRIBUTIONS

**Yifan Lu:** Conceptualization (equal); formal analysis (lead); investigation (lead); methodology (lead); writing – original draft (lead). **Ali Hajjar:** Formal analysis (supporting); investigation (equal); validation (lead). **Vincent Cryns:** Resources (equal); writing – review and editing (equal). **Amy Trentham‐Dietz:** Funding acquisition (equal); resources (equal); writing – review and editing (equal). **Ronald E. Gangnon:** Resources (equal); writing – review and editing (equal). **Brandy Heckman‐Stoddard:** Writing – review and editing (supporting). **Oguzhan Alagoz:** Conceptualization (equal); funding acquisition (equal); project administration (lead); supervision (lead); writing – original draft (equal).

## FUNDING INFORMATION

This work was supported by National Cancer Institute grants U01 CA253911 and P30 CA014520.

## CONFLICT OF INTEREST

OA has been a paid consultant for Bristol Myers Squibb, Johnson & Johnson, and Exact Sciences, outside of the submitted work. No other conflicts to report.

## Supporting information


Figure S1.

## Data Availability

Computer code: Available from Dr. Alagoz (email, alagoz@engr.wisc.edu). Analytic dataset: Available from Dr. Alagoz (email, alagoz@engr.wisc.edu).
