# Supplementary material for: Breast cancer risk for women with diabetes and the impact of metformin: A meta‐analysis
Source: Cancer Med. 2022 Dec 19;12(10):11703–18. doi: 10.1002/cam4.5545 (PMC10242307; doi:10.1002/cam4.5545)
Supplement: Supplementary file 1 — Figure S1. [file CAM4-12-11703-s001.docx]

**Appendix**

**Table of contents**

[**Appendix Figure 1: Funnel plot of the studies included in the meta-analysis estimating the relative risk of breast cancer for women with type 2 diabetes** 2](#_Toc98531429)

[**Appendix Figure 2: Summary relative risk of the studies included in the meta-analysis estimating the relative risk of breast cancer for women with type 2 diabetes according to geographic location** 3](#_Toc98531430)

[**Appendix Figure 3: Subgroup analysis for the meta-analysis estimating the relative risk of breast cancer for women with type 2 diabetes** 4](#_Toc98531431)

[**Appendix Figure 4: Funnel plots of the studies included in the meta-analysis estimating the relative risk of breast cancer for women with type 2 diabetes who use metformin** 5](#_Toc98531432)

[**Appendix Figure 5: Summary relative risk of the studies included in the meta-analysis estimating the relative risk of breast cancer for women with type 2 diabetes by the year of publication** 6](#_Toc98531433)

[**Appendix Table 1: Combination of search terms** 7](#_Toc98531434)

[**Appendix Table 2: Summary of subgroup analysis for the meta-analysis estimating the relative risk of breast cancer for women with type 2 diabetes** 8](#_Toc98531435)

# **Appendix Figure 1: Funnel plot of the studies included in the meta-analysis estimating the relative risk of breast cancer for women with type 2 diabetes**


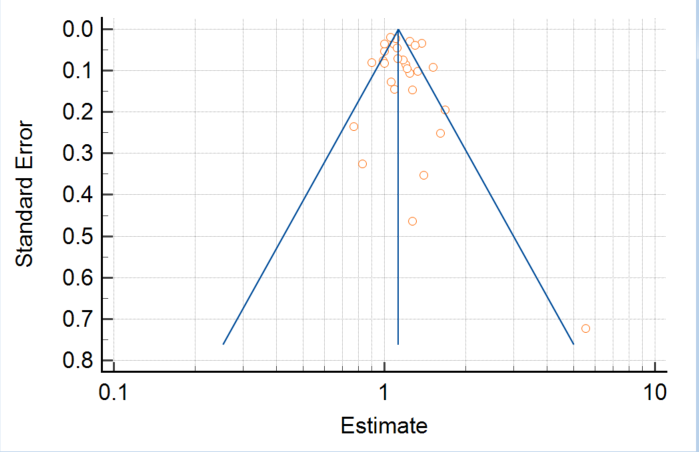


# **Appendix Figure 2: Summary relative risk of the studies included in the meta-analysis estimating the relative risk of breast cancer for women with type 2 diabetes according to geographic location**


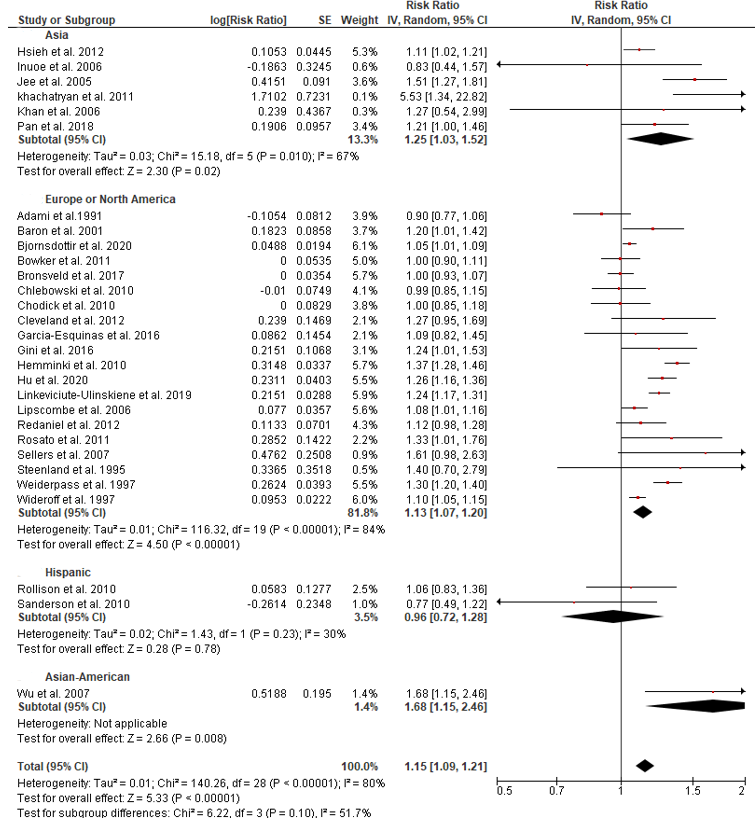


# **Appendix Figure 3: Subgroup analysis for the meta-analysis estimating the relative risk of breast cancer for women with type 2 diabetes**


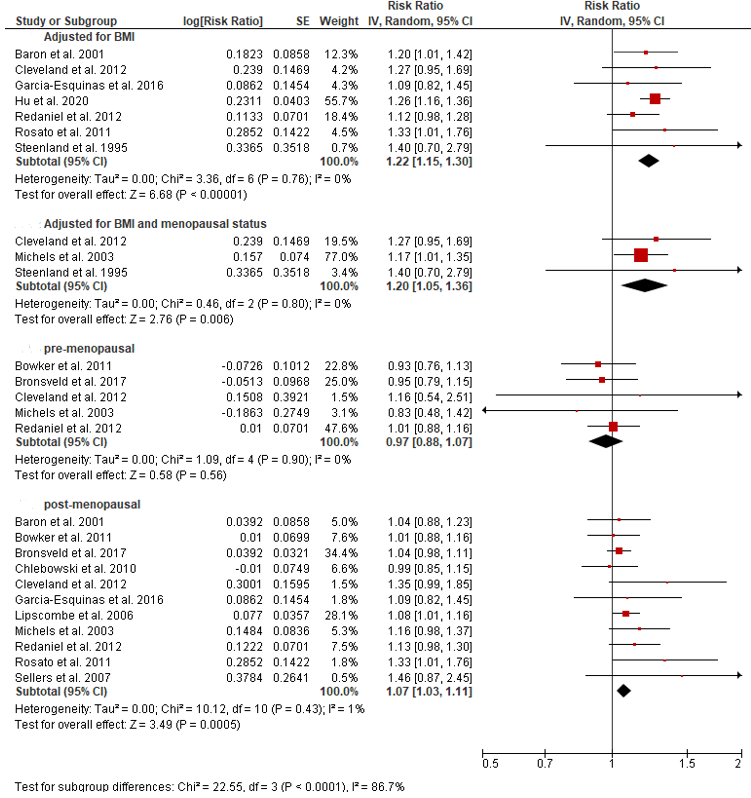


# **Appendix Figure 4: Funnel plots of the studies included in the meta-analysis estimating the relative risk of breast cancer for women with type 2 diabetes who use metformin**


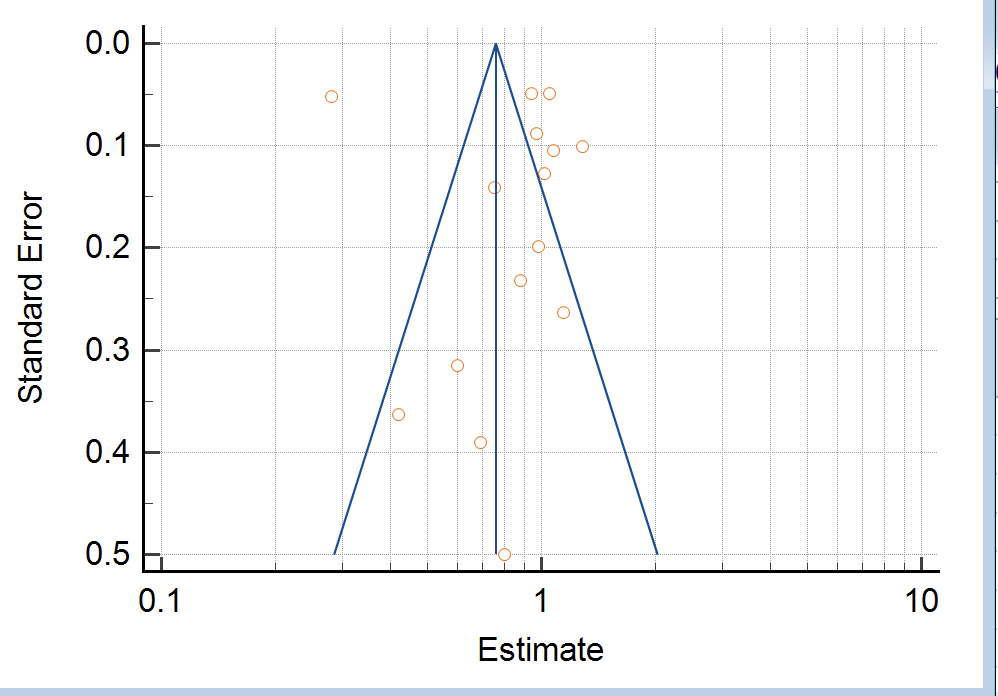


# **Appendix Figure 5: Summary relative risk of the studies included in the meta-analysis estimating the relative risk of breast cancer for women with type 2 diabetes by the year of publication**


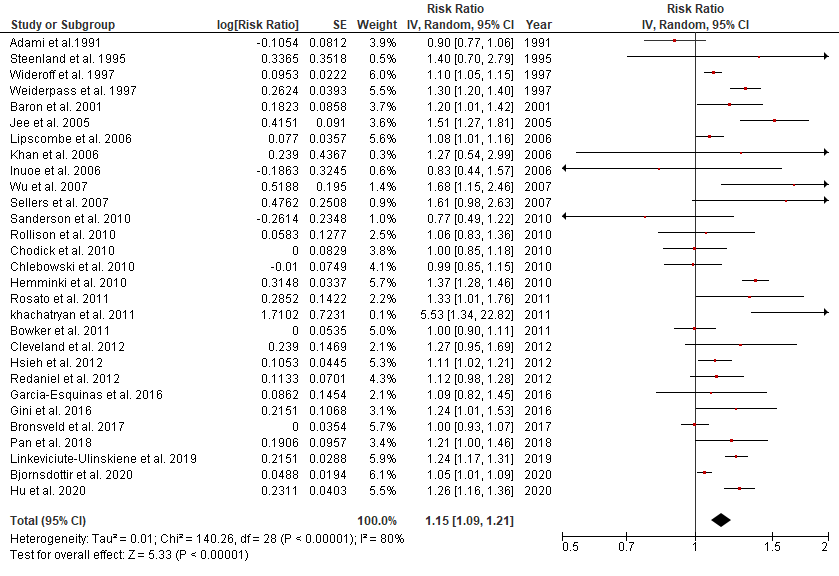


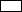

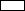

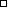

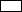


# **Appendix Table 1: Combination of search terms**

| Database | Meta-analysis | Search term inputs |
| --- | --- | --- |
| PubMed | The risk of breast cancer only among women with type 2 diabetes | (“breast neoplasms”[mesh] OR ((neoplasms[tiab] OR neoplasm[tiab] OR tumor[tiab] OR tumors[tiab] OR cancer[tiab] OR cancers[tiab] OR carcinoma[tiab] OR carcinomas[tiab]) AND (breast[tiab] OR breasts[tiab] OR mammary[tiab]))) AND (“diabetes mellitus”[mesh] OR diabetes[tiab] OR diabetic[tiab] OR “wolfram syndrome”[tiab] OR “Donohue syndrome”[tiab] OR “prediabetic state”[tiab]) |
|  | The impact of the use of metformin on breast cancer risk among women with type 2 diabetes | (“breast neoplasms”[mesh] OR ((neoplasms[tiab] OR neoplasm[tiab] OR tumor[tiab] OR tumors[tiab] OR cancer[tiab] OR cancers[tiab] OR carcinoma[tiab] OR carcinomas[tiab]) AND (breast[tiab] OR breasts[tiab] OR mammary[tiab]))) AND (“diabetes mellitus”[mesh] OR diabetes[tiab] OR diabetic[tiab] OR “wolfram syndrome”[tiab] OR “Donohue syndrome”[tiab] OR “prediabetic state”[tiab]) AND (metformin[mesh] OR metformin[tiab] OR riomet[tiab] OR glumetza[tiab] OR Dimethylbiguanidine[tiab] OR Dimethylguanylguanidine[tiab] OR Glucophage[tiab] OR fortamet[tiab]) |
| Scopus | The risk of breast cancer only among women with type 2 diabetes | ("breast neoplasms" OR ((neoplasms OR neoplasm OR tumor OR tumors OR cancer OR cancers OR carcinoma OR carcinomas) AND (breast OR breasts OR mammary))) AND ("diabetes mellitus" OR diabetes OR diabetic OR "wolfram syndrome" OR "Donohue syndrome" OR "prediabetic state") |
|  | The impact of the use of metformin on breast cancer risk among women with type 2 diabetes | ("breast neoplasms" OR ((neoplasms OR neoplasm OR tumor OR tumors OR cancer OR cancers OR carcinoma OR carcinomas) AND (breast OR breasts OR mammary))) AND ("diabetes mellitus" OR diabetes OR diabetic OR "wolfram syndrome" OR "Donohue syndrome" OR "prediabetic state") AND (metformin OR metformin OR riomet OR glumetza OR dimethylbiguanidine OR dimethylguanylguanidine OR glucophage OR fortamet) |

# **Appendix Table 2: Summary of subgroup analysis for the meta-analysis estimating the relative risk of breast cancer for women with type 2 diabetes**

| Subgroups (number of studies) | RR/OR/HR (95% CI) |
| --- | --- |
| With adjustment for BMI (7) [33, 47, 48, 72, 75, 80, 81] | 1.22 (1.15-1.30) |
| With adjustment for BMI and menopausal status (3) [34, 72, 81] | 1.20 (1.05-1.36) |
| Pre-menopausal (5) [34, 47, 50, 62, 79] | 0.97 (0.88-1.07) |
| Post-menopausal (10) [34, 45, 47, 48, 50, 62, 75, 76, 79-81] | 1.07 (1.03-1.11) |
